# Carboxylated Poly-l-Lysine as a Macromolecular Cryoprotective Agent Enables the Development of Defined and Xeno-Free Human Sperm Cryopreservation Reagents

**DOI:** 10.3390/cells10061435

**Published:** 2021-06-08

**Authors:** Hiroki Takeuchi, Mikiko Nishioka, Tadashi Maezawa, Yuko Kitano, Kento Terada-Yoshikawa, Ryota Tachibana, Manabu Kato, Suong-hyu Hyon, Yuki Gen, Kayo Tanaka, Kuniaki Toriyabe, Masafumi Nii, Eiji Kondo, Tomoaki Ikeda

**Affiliations:** 1 Department of Obstetrics and Gynecology, Mie University Graduate School of Medicine, Tsu 514-8507, Japan; m-nishioka@clin.medic.mie-u.ac.jp (M.N.); yoshikawa-k@clin.medic.mie-u.ac.jp (K.T.-Y.); r-tachibana@clin.medic.mie-u.ac.jp (R.T.); tanaka-ky@clin.medic.mie-u.ac.jp (K.T.); t-kuniaki@clin.medic.mie-u.ac.jp (K.T.); m-nii1984@clin.medic.mie-u.ac.jp (M.N.); eijikon@clin.medic.mie-u.ac.jp (E.K.); t-ikeda@clin.medic.mie-u.ac.jp (T.I.); 2 Center of Advanced Reproductive Medicine, Mie University Hospital, Tsu 514-8507, Japan; tada-m@clin.medic.mie-u.ac.jp (T.M.); y-kitano@clin.medic.mie-u.ac.jp (Y.K.); katouuro@clin.medic.mie-u.ac.jp (M.K.); 3 Department of Obstetrics and Gynecology, Mie University Hospital, Tsu 514-8507, Japan; 4 Department of Nephro-Urologic Surgery and Andrology, Mie University Graduate School of Medicine, Tsu 514-8507, Japan; 5 BMG Inc., Kyoto 601-8023, Japan; biogen@bmg-inc.com; 6 BioVerde Inc., Kyoto 601-8023, Japan; ygen_bioverde@bmg-inc.com

**Keywords:** xeno-free and defined, cryopreservation reagent, low-molecular-weight cryoprotective agents, macromolecular-weight cryoprotective agents, saccharides

## Abstract

In human sperm cryopreservation, test yolk buffer and human serum albumin have been used as permeating macromolecular-weight cryoprotectants. In clinical reproductive medicine, human serum albumin is frequently used because of low risks of zoonoses and allergic reactions. However, the risk of allogeneic infectious diseases exists, and the supply may be unstable because human serum albumin is derived from human blood. Therefore, the development of xeno-free human sperm cryopreservative reagents that could overcome the aforementioned problems is warranted. We succeeded in developing a new xeno-free and defined sperm cryopreservation reagent containing glycerol, carboxylated poly-l-lysine, and raffinose. The cryopreservation reagent was not significantly different in terms of sperm motility, viability, and DNA fragmentation and was comparable in performance to a commercial cryopreservation reagent containing human serum albumin. Moreover, the addition of saccharides was essential for its long-term storage. These results may help elucidate the unknown function of macromolecular-weight permeating cryoprotective agents.

## 1. Introduction

Spermatozoal cryopreservation technology (SCPT) provides the advantage of storing sperms semi-permanently for future use [1]. This technology is widely used to preserve genetic resources, such as those of endangered, rare [2], and laboratory animals [3,4], as well as for the efficient production of domestic animals [5] and applications associated with assisted reproductive technology (ART) [6]. Dramatic advances in ART and the development of numerous related technologies have been reported recently [7,8,9,10]. Specifically, SCPT has drastically altered the significance and efficiency of ART via intracytoplasmic sperm injection [7], a technique that enables fertilization with only a single spermatozoon. A priori cryopreservation of spermatozoa is important for patients with severe oligozoospermia, asthenozoospermia, and teratozoospermia [11].

Suppression of cytoplasmic and extracellular ice crystal formation during freezing and blocking of cell swelling due to osmotic pressure during thawing are crucial to cryopreservation of cells, including sperms [12]. Low-molecular-weight (Mw) permeating cryoprotective agents (CPAs), including glycerol (Glyc), dimethyl sulfoxide (DMSO), ethylene glycol (EG), and propylene glycol (PG), can effectively suppress cytoplasmic ice crystal formation [13]. However, these agents are potentially cytotoxic and should be used at low concentrations [14,15]. Consequently, several low-Mw CPAs with low cytotoxicity have been developed [16]. Of these CPAs, Glyc yields the highest motility rate after freeze–thawing and is widely used in human sperm cryopreservation. Moreover, the addition of amides and saccharides such as sucrose is essential to prevent the swelling of cells induced by osmotic pressure, as well as the edema induced by low-Mw CPAs during thawing [12,17]. Therefore, non-permeating macro-Mw CPAs, such as test yolk buffer (TYB) [18,19] and human serum albumin (HSA) [20], should be added to the cryopreservation medium to prevent intracellular ice crystal formation induced by extracellular ice crystal formation.

TYB and HSA have been used as macro-Mw CPAs for human sperm cryopreservation. However, TYB has risks of zoonoses and allergic reactions. Thus, HSA is most commonly used as a macro-Mw CPA in current applications, although its supply may be unstable due to variations between lots and risks of allogeneic infections. Therefore, the development of a xeno-free human sperm cryopreservative reagent that could overcome the aforementioned problems is expected.

Hydroxypropyl cellulose (HPC) is a polysaccharide of variable length that exhibits physical properties similar to those of HSA, which forms viscous gels at low temperatures. It is also used as an excipient in food additives and drugs and has guaranteed safety [21]. It has also been used to freeze human oocytes, embryos, and blastocysts, and has shown good results [22,23].

Carboxylated poly-l-lysine (CPLL), an ampholytic polymer compound, is reportedly less cytotoxic than DMSO [24]. This compound has high cryoprotective properties and is associated with membrane protection [24,25,26,27,28,29]. Several studies reported that CPLL had been used to cryopreserve cells [24,28,30,31,32,33,34] and embryos [35,36]. Although two studies reported the cryopreservation of bovine sperm using CPLL [37,38], to the best of our knowledge, no study has explored this reagent in human samples, particularly with respect to sperm cryopreservation. Additionally, clinically used sperm cryopreservation reagents are often developed using sperms from healthy subjects and may not function as described in the paper.

The purpose of this study was to identify macro-Mw CPAs that could serve as alternatives to TYB or HSA for clinical use. We subsequently aimed to examine low-Mw CPAs and saccharides that act effectively with the macro-Mw CPA identified in this study. Finally, we developed a clinically defined and xeno-free cryopreservation reagent and investigated its effect on sperm.

## 2. Materials and Methods

### 2.1. Ethical Approval

Informed consent for this prospective study was obtained from all participants who donated semen. The sperm samples used in this study were collected from 105 male infertile patients in our university hospital between 2016 and 2020 and were either discarded after the semen analysis or retained after the completion of in vitro fertilization (IVF). Fujifilm Irvine’s cryopreservation reagent (Fujifilm Irvine Scientific, Santa Ana, CA, USA) is routinely used in our clinical practice; therefore, this reagent was used for all comparisons in our study.

### 2.2. Cryopreservation Reagents

To develop new human sperm cryopreservation reagents, low-Mw and macro-Mw CPAs and saccharides were added at various concentrations based on the modified human tubal fluid (HTF) medium (Fujifilm Irvine Scientific). Glyc, EG, PG, and lactamide (Lac) were used as low-Mw CPAs, whereas CPLL (–COOH: 65 mol%, Mw: 4000–5000, provided by BioVerde, Kyoto, Japan) and HPC (Nissoe Co., Ltd., Tokyo, Japan) were used as macro-Mw CPAs. Fructose (Fru), sucrose (Suc), trehalose (Tre), and raffinose (Raf) (Nacalai Tesque, Kyoto, Japan) were used as saccharides. The final concentrations of the low- and macro-Mw CPAs and saccharides when mixed with sperm were 2–10% *v*/*v* or *w*/*v*, 0.05–20% *w*/*v*, and 0.05–0.5 M, respectively (Table 1). For all the experiments, the cryopreservation reagent was mixed with the sperm suspension in the ratio of 400 µL/100 µL. A sperm maintenance medium (99176, discontinued, Fujifilm Irvine Scientific) was used in the control group and prepared according to the manufacturer’s protocol; the final volume was 500 µL. The sperm maintenance medium included sodium and calcium lactate and N-2-hydroxyethyl piperazine-N-2-ethanesulfonic acid (HEPES) in addition to Glyc, HSA, Suc, and glucose.

### 2.3. Study Design

**Figure 1:**
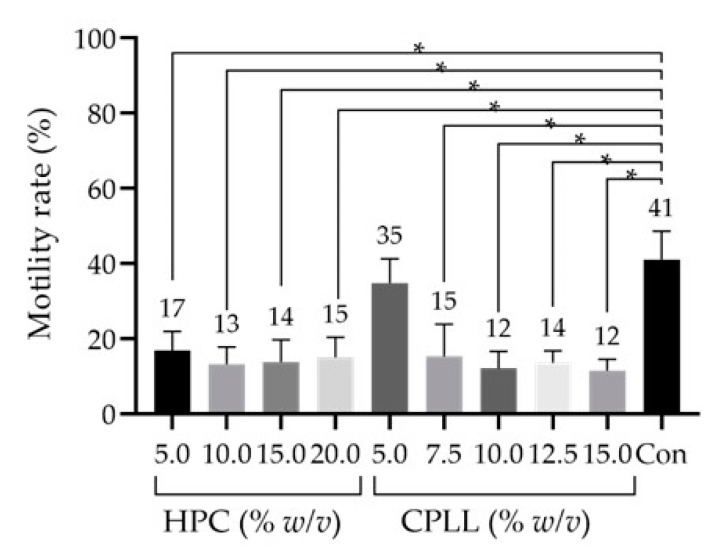
Sperm motility rates after freeze–thawing with cryopreservation reagents containing macro-molecular-weight (Mw) cryoprotective agents (CPAs) at different concentrations. Hydroxypropyl cellulose (HPC), 5.0–20.0% *w*/*v*. Carboxylated poly-l-lysine (CPLL), 5.0–15.0% *w*/*v*. The low-Mw CPA was 4% *v*/*v* glycerol (Glyc), and no additional saccharides were used. Dunnett’s test was performed to compare motility rates between the cryopreserved sperm samples. Data are expressed as the means ± standard deviations (*n* = 6); *p*-values < 0.05 were considered statistically significant. Asterisks (*) indicate a statistically significant difference; Con, commercial cryopreservation reagent (Fujifilm Irvine).

**Figure 2:**
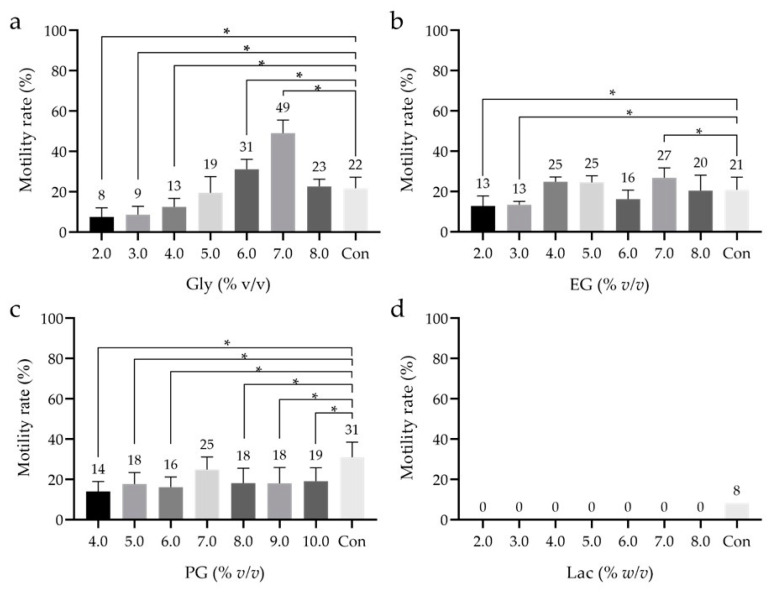
Sperm motility rate after freeze–thawing with cryopreservation reagents containing low-Mw cryoprotective agents (CPAs) at different concentrations. (**a**) Glycerol (Glyc). (**b**) Ethylene glycol (EG). (**c**) Propylene glycol (PG). (**d**) Lactamide (Lac). The motility of sperms with Lac could not be confirmed under all conditions. Thus, the measurement of Lac was performed only once. CPLL at 5% *w*/*v* was used as the macro-Mw CPA, and no additional saccharides were used. Dunnett’s test was performed to compare sperm motility rates after freeze–thawing with that of the control; *p*-value < 0.05 was considered statistically significant. The data are expressed as the means ± standard deviations (**a**–**c**; *n* = 9, d; *n* = 1). Asterisks (*) indicate a statistically significant difference; low-Mw CPA, low-molecular-weight cryoprotective agent; macro-Mw CPA, macromolecular-weight cryoprotective agent; Con, commercial cryopreservation reagent (Fujifilm Irvine).

**Figure 3:**
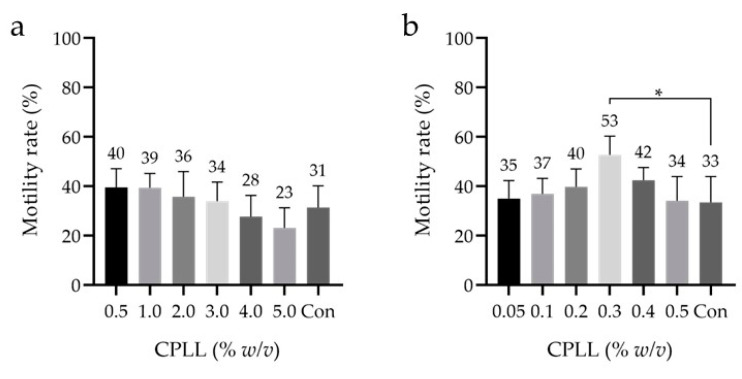
Sperm motility rates after freeze–thawing with cryopreservation reagents containing different concentrations (≤ 5% *w*/*v*) of the macro-Mw CPA CPLL. (**a**) The concentrations of CPLL ranged from 0.5% to 5% *w*/*v*. (**b**) The concentrations of the macro-Mw CPA CPLL ranged from 0.05% to 0.5% *w*/*v* with 7% *v*/*v* Glyc as the low-Mw CPA and no additional saccharides used. Dunnett’s test was performed; *p*-value < 0.05 was considered statistically significant. The data are expressed as the means ± standard deviations (*n* = 9). Asterisks (*) indicate a statistically significant difference; low-Mw CPA, low-molecular-weight cryoprotective agent; macro-Mw CPA, macromolecular-weight cryoprotective agent; Con, commercial cryopreservation reagent (Fujifilm Irvine).

**Figure 4:**
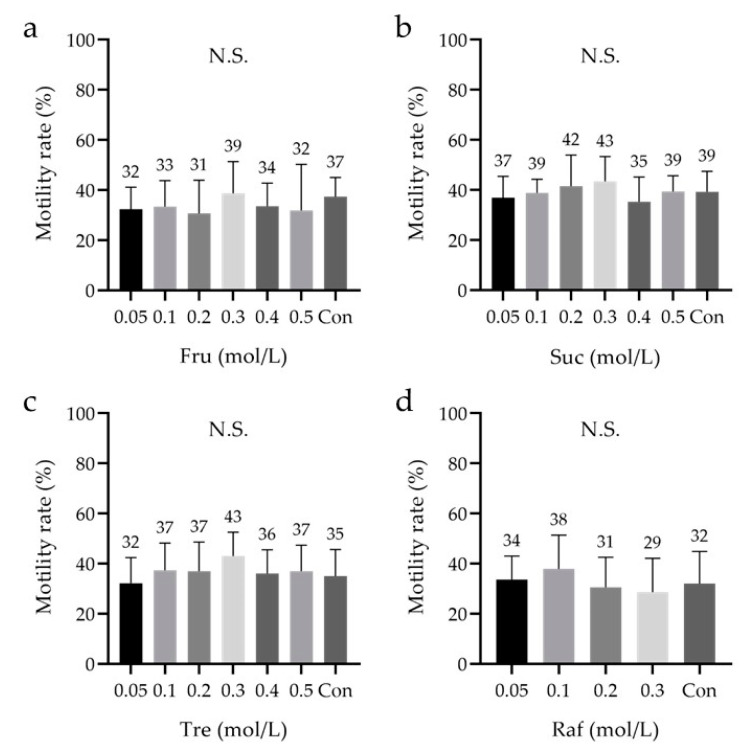
Sperm motility rate after freeze–thawing with cryopreservation reagents containing saccharides at different concentrations. (**a**). Fructose (Fru), 0.05–0.5 M. (**b**) Sucrose (Suc), 0.05–0.5 M. (**c**) Trehalose (Tre), 0.05–0.5 M. (**d**) Raffinose (Raf), 0.05–0.3 M. (**a**–**d**) The low-Mw CPA was 7% *v*/*v* glycerol (Glyc), and the macro-Mw CPA was 0.3% *w*/*v* carboxylated poly-l-lysine (CPLL). The cryopreservation reagents were compared, and the concentration of each saccharide that yielded the highest motility rate was determined (**a**–**d**). Dunnett ’s test was performed. The data are expressed as the means ± standard deviations (n = 9); low-Mw CPA, low-molecular-weight cryoprotective agent; macro-Mw CPA, macromolecular-weight cryoprotective agent; Con, commercial cryopreservation reagent (Fujifilm Irvine); N.S, not significant.

**Figure 5:**
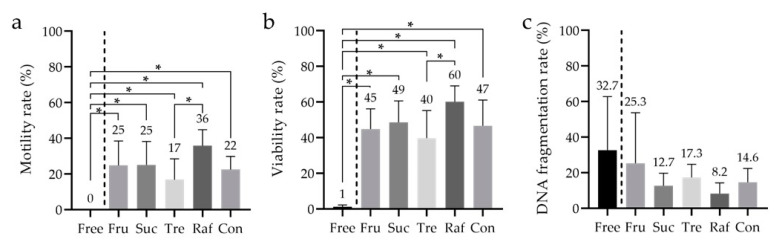
Sperm motility, viability, and DNA fragmentation rates after freeze–thawing with cryopreservation reagents containing different saccharides. (**a**) The motility rate was analyzed to determine the sperm cryopreservation reagent that yielded the best conditions, including the saccharides explored in Figure 4. (**b**) The viability was analyzed using the sperm viability test (SVT) with residual samples from Figure 5a. (**c**) The DNA fragmentation rate was analyzed using flow cytometry with residual samples from Figure 5a. (**a**) The low-Mw CPA was 7% *v*/*v* glycerol (Glyc), and the macro-Mw CPA was 0.3% *w*/*v* carboxylated poly-l-lysine (CPLL). Tukey’s multiple comparison test was performed in all groups except ‘Free; *p*-value < 0.05 was considered statistically significant. The data are expressed as the means ± standard deviations (*n* = 8). Asterisks (*) indicate a statistically significant difference; Free, no saccharides; Fru, fructose; Suc, sucrose; Tre, trehalose; Raf, raffinose; low-Mw CPA, low-molecular-weight cryoprotective agent; macro-Mw CPA, macromolecular-weight cryoprotective agent; Con, commercial cryopreservation reagent (Fujifilm Irvine).

**Figure 6:**
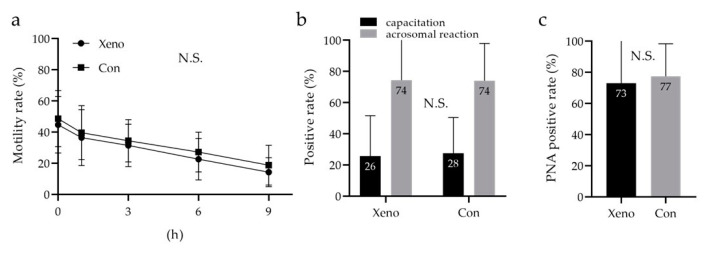
Quantitative analysis of sperm motility with a longevity curve, capacitation status, and acrosome reaction. (**a**) Quantitative motility of sperm with longevity was assessed using Makler counting chamber measurements every 0, 1, 3, 6, and 9 h. (**b**) Capacitation status and acrosome reaction assessed by chlortetracycline (CTC)/Hoechst 33258 (H258) imaging under a fluorescence microscope. (**c**) Assessment of the acrosome reaction with lectin from *Arachis hypogaea* (peanut) conjugated FITC and Ethidium Homodimer-1 using flow cytometry. Two-way repeated measures ANOVA was performed in (**a**), Mann–Whitney *U* test—in (**b**,**c**). The data are expressed as the means ± standard deviations (*n* = 9). Xeno: the developed xeno-free cryopreservation reagent consisting of a low-Mw CPA (7% *v*/*v* Glyc), a macro-Mw CPA (0.3% *w*/*v* CPLL), and a saccharide (0.3 M Raf); low-Mw CPA, low-molecular-weight cryoprotective agent; macro-Mw CPA, macromolecular-weight cryoprotective agent; Con, commercial cryopreservation reagent (Fujifilm Irvine); N.S, no significant.

The procedure followed in this study is detailed in Table 1. For the first experiment, the optimal macro-Mw CPA was chosen from HPC and CPLL, and each patient’s sample was divided in 10 parts; this procedure was performed for six patients (Figure 1). This cryopreservation reagent contained 4% *v*/*v* Glyc and no saccharide in the HTF medium (Fujifilm Irvine Scientific). In the second experiment, the optimal low-Mw CPA was chosen from Glyc, EG, PG, and Lac, and its concentration was determined. For Glyc, EG, PG, and Lac, each patient’s sample was divided in eight parts; Glyc, EG, and PG were used in nine patients, Lac—in one patient (Figure 2). This cryopreservation reagent contained 5% *w*/*v* CPLL and no saccharide in the HTF medium. In the third experiment, to examine the optimum concentration of CPLL, each patient’s sample was divided in seven parts, and the procedure was performed for nine patients (Figure 3). Since the number of CPLL concentrations to be determined in this experiment was large, the evaluation was divided in two batches: 0.05–0.5% *w*/*v* and 0.5–5% *w*/*v*. This cryopreservation reagent contained 7% *v*/*v* Glyc and no saccharide in the HTF medium. In the fourth experiment, we investigated the optimal saccharide from among Fru, Suc, Tre, and Raf and its concentration. For Fru, Suc, and Tre, each patient’s sample was divided in seven parts, and for Raf, each patient’s sample was divided in five parts; each procedure was performed for nine patients (Figure 4). This cryopreservation reagent contained 7% *v*/*v* Glyc and 0.3% *w*/*v* CPLL in the HTF medium. In the fifth experiment, we investigated the motility, viability and sperm DNA fragmentation (SDF) in each saccharide at the optimized concentrations determined in Figure 4 and compared them with each other, including those without a saccharide. This experiment was performed for eight patients, and each patient’s sample was divided in six parts. These samples were thawed, washed, and divided in three aliquots to measure the motility, viability and SDF rate (Figure 5). This cryopreservation reagent contained no saccharide, 0.3 M Fru, 0.3 M Suc, 0.3 M Tre, or 0.1 M Raf, with 7% *v*/*v* Glyc and 0.3% *w*/*v* CPLL in the HTF medium. In the final experiment, samples were obtained from nine patients, and each patient’s sample was divided in two parts. These samples were thawed, washed, and divided in three aliquots for the quantitative analysis of sperm motility with a longevity curve and assessment of the capacitation status and acrosome reaction. This cryopreservation reagent contained 7% *v*/*v* Glyc, 0.3% *w*/*v* CPLL, and 0.1 M Raf in the HTF medium (Figure 6).

### 2.4. Sperm Preparation, Cryopreservation, and Thawing

Semen was collected by masturbation and immediately processed for semen analysis and density gradient centrifugation (DGC) after liquefaction. IVF samples were treated with DGC + swim-up after ejaculation and stored in an incubator at 37 °C, 5% CO_2_, and 5% O_2_ until IVF was completed. The liquefied semen underwent DGC using a 50% and a 90% isolate (Fujifilm Irvine Scientific). Centrifugation was performed for 15 min at 400× *g* according to the DGC method. Subsequently, the washed sperms were centrifuged using a multipurpose handling medium (MHM) containing 10% *v*/*v* serum substitute supplement (SSS; Fujifilm Irvine Scientific) for 10 min at 400× *g*. HTF containing 10% *v*/*v* SSS was added to the washed sperms, and the suspended sperm cells were measured using a Makler counting chamber (Sefi Medical Instruments, Haifa, Israel). Sperm concentration and motility rate were calculated as described by Bjorndahl et al. [39]. To evaluate at least 200 spermatozoa, all the spermatozoa within 100 squares were counted in each of the overlapping assessments. Suspended sperm samples with a motility rate >80% and total sperm concentration >40 million/mL were used in subsequent cryopreservation experiments. For clinical IVF, the motility cutoff value is >90%. However, in this study, the cutoff value was set at >80% as it was difficult to collect samples with >90% motility. In practice, 17 samples had motility <90% (thirteen samples were ≥85% and <90%, four samples were ≥80% and <85%) (Supplementary Tables S1 and S2).

Each sperm suspension was mixed with the cryopreservation reagent in cryotubes in the 1:4 ratio. Cryotubes were used for three reasons. First, they were recommended as control by the manufacturer of the cryopreservation reagent. Second, we are well-accustomed with their use in clinical practice. Third, we had examined the same control reagent while using cryostraws in another study and had observed results worse than those achieved using cryotubes. The tubes were placed 3 cm above the liquid nitrogen surface for 10 min using a frozen dedicated floating device (Kitazato, Tokyo, Japan), followed by storage of the tubes in liquid nitrogen. The cryostorage was <24 h for the motility test but more than 3–4 weeks for the sperm viability and DNA fragmentation tests.

The frozen samples were thawed in a 37 °C water bath until just before the ice melted (2–3 min). Subsequently, they were suspended in 5 mL of 10% *v*/*v* SSS/MHM warmed to 37 °C and centrifuged for 5 min at 400× *g*. The supernatant was removed and suspended in 100 μL of 10% *v*/*v* SSS/MHM, and the sperms were counted using a Makler counting chamber.

### 2.5. Sperm Viability Test (SVT)

The viability tests were performed on eight samples with blinding. MHM was added, and the sperm suspension was divided in three aliquots to measure motility and SVT and SDF rates and centrifuged for 5 min at 400× *g*. After discarding the supernatant, 1 mL phosphate-buffered saline (PBS) (–) containing 0.1% *w*/*v* bovine serum albumin (Nacalai Tesque) was added to the washed samples, and sperm viability was analyzed using a BD cell viability kit (Becton Dickinson, Franklin Lakes, NJ, USA) according to the manufacturer’s protocol. Moreover, 1.0 µL of each dye solution (i.e., thiazole orange and propidium iodide, Becton Dickinson) was added to 1 mL sperm suspension, and the mixture was incubated for 5 min. Fluorescence intensities were analyzed using a BD FACSCanto II flow cytometer with the FACSDiva software (Becton Dickinson). Negative SVT samples were defined as dead cells fixed with 4% *w*/*v* paraformaldehyde (PFA; Nacalai Tesque) for 20 min at room temperature.

### 2.6. SDF Analysis Using the TUNEL Assay

Freeze–thawed samples were prepared in the same manner as for the SVT. These samples were tested for SDF using a terminal deoxynucleotidyl transferase dUTP nick end labeling (TUNEL) assay as directed by the MEBSTAIN Apoptosis TUNEL Kit Direct (MBL Life Science, Nagoya, Japan). The TUNEL assay was performed according to the method described by Sharma et al. [40]. Briefly, the washed samples were adjusted to a density of <1 million sperm cells/mL and fixed with 1 mL 4% *w*/*v* PFA for 30 min at 4 °C. The supernatant of the PFA was discarded after centrifugation at 1500× *g* for 5 min. Each pellet was resuspended with 1 mL ice-cold ethanol (70% *v*/*v*) and incubated for 30 min at −20 °C. After centrifugation at 1500× *g* for 5 min, the supernatant was discarded and the samples were washed twice with 0.2% *w*/*v* BSA/PBS (–) (Nacalai Tesque). A staining solution was prepared according to the manufacturer’s protocol, and the stained samples were incubated for 60 min at 37 °C in the dark. After rinsing with 0.2% *w*/*v* BSA/PBS (–), the samples were incubated with 5 µg/mL propidium iodide solution (Nacalai Tesque) for 30 min in the dark. Fluorescence intensities were analyzed using a BD FACSCanto II flow cytometer with the FACSDiva software to determine the percentage of sperm cells with DNA fragmentation, and a minimum of 5000 events were recorded. Negative and positive controls were prepared and subjected to all analyses (*n* = 8). TUNEL-positive sperms were defined as those containing fragmented DNA after 20-min DNase I treatment at room temperature (Nippon Gene, Tokyo, Japan).

### 2.7. Quantitative Analysis of Sperm Motility with a Longevity Curve

The sample was thawed, washed, and subsequently divided in three aliquots, which were used for quantitative analysis of sperm motility with a longevity curve and assessment of the capacitation status and acrosome reaction.

Quantitative assessment of motility was performed using Makler counting chamber measurements every 0, 1, 3, 6, and 9 h. The washed sperms were suspended in 200 µL of 10% SSS/HTF and incubated at 37 °C, 5% CO_2_, and 5% O_2_.

### 2.8. Assessment of the Capacitation Status

Capacitation status of the sperm was assessed using the CTC fluorescence assay method according to the methods described by Kong et al. [41]. CTC was used to judge the capacitation status of the sperm and Hoechst 33258 (H258) was used to confirm the living/dead state of sperms. Briefly, a CTC stock solution was prepared: 130 mM NaCl, 5 mM cysteine, and 750 μM CTC in a 20 mM Tris-HCl buffer (pH 7.8) (all Nacalai Tesque). This solution was stored in the dark at 4 °C just before use. We added 100 μL of 10 μg/mL H258 to the washed sample, and a 5-μL aliquot of the treated sperm suspension was placed on a slide warmed to 37 °C. Next, 5 μL CTC stock solution was added, and within 30 s, 0.5 μL 2% glutaraldehyde (Nacalai Tesque) in 1 M Tris buffer (pH 7.8) was added. The slides were evaluated with a confocal laser scanning microscope (FV1000; Olympus, Tokyo, Japan). In each sample, 200 live cells were assessed for CTC staining patterns. Surviving sperms that were not stained with H258 were selected and three CTC fluorescence patterns were identified: F, whole sperm head stained, incapacitated sperm; B, only the acrosome stained, sperm that achieved or is achieving capacitation; AR, only the equatorial zone stained, acrosomal reactive sperm. The capacitation rate was defined as the ratio of B patterns in the total of 200 sperms.

### 2.9. Assessment of the Acrosome Reaction

Assessment of the acrosomal status was performed using lectin from *Arachis hypogaea* (peanut) conjugated FITC (PNA-FITC; Sigma-Aldrich, St. Louis, MO, USA) and Ethidium Homodimer-1 (EthD-1; Setareh Biotech, Eugene, OR, USA), according to the methods described by Szasz et al. [42]. PNA-FITC was used to assess the outer plasma membrane and acrosome integrity of the sperm, whereas EthD-1 was used to confirm the living/dead state of sperms. Briefly, the washed samples were adjusted to a density of <1 million sperm cells/mL and labelled with 10 µg/mL PNA-FITC and 1 µM EthD-1 for 10 min at 37 °C. Fluorescence intensities were analyzed using a BD FACSCanto II flow cytometer with the FACSDiva software. The data for 10,000 sperm events were collected per analysis. The ratio of FITC-PNA-stained sperms in live sperm samples (not stained with EthD-1) was calculated.

### 2.10. Statistical Analysis

All the data are expressed as the means ± standard deviations. Statistical analysis was performed using GraphPad Prism 8 (GraphPad Software, La Jolla, CA, USA). Dunnett’s test was used to compare motility of the freeze–thawed sperms with that of the control (Figure 1, Figure 2, Figure 3 and Figure 4). Tukey’s multiple comparison test was used to compare the effects of saccharides, as well as the motility, viability, and DNA fragmentation rates of the cryopreserved sperms in all the groups (Figure 5). Two-way repeated measures analysis of variance (ANOVA) was used to compare the quantitative motility of sperms with longevity between the developed cryopreservation reagent and a commercial reagent, and Mann−Whitney *U* test was used to compare the capacitation status and acrosome reaction between the control and the developed cryopreservation reagents. The results were considered statistically significant at *p*-value < 0.05.

## 3. Results

### 3.1. Effect of Macro-Mw CPAs

Cryopreservation reagents require a combination of low-Mw permeating CPAs and non-permeating macro-Mw CPA; however, low- and macro-Mw CPAs cannot be examined independently. To evaluate a macro-Mw CPA, the low-Mw CPA used was 4% *v*/*v* Glyc, as reported by Fujita et al., since high concentrations of Glyc are cytotoxic to sperm [17]. To investigate the effects of macro-Mw CPAs on sperm cryopreservation, macro-Mw CPAs (HPC (5%, 10%, 15%, 20% *w*/*v*) and CPLL (5%, 7.5%, 10%, 12.5%, 15% *w*/*v*)) were added at each indicated final concentration. These concentrations are commonly used in human and bovine experiments. Subsequently, the sperm motility rate was measured after freeze–thawing (Figure 1). After washing with 10% *v*/*v* SSS/MHM, sperm motility was significantly reduced in all the thawed samples treated with different concentrations of HPC and CPLL except for 5% *w*/*v* CPLL as compared with that of the control. Sperm motility tended to be the highest in the samples treated with 5% *w*/*v* CPLL; hence, we examined 5% *w*/*v* CPLL and low-Mw CPAs in detail.

### 3.2. Effect of Low-Mw CPAs

Examination of macro-Mw CPAs revealed no significant differences in sperm motility after freeze–thawing. We considered it necessary to evaluate the effects of choosing a low Mw-CPA before reinvestigating the concentration of macro-Mw CPAs. Glyc at 6% or 7% *v*/*v* yielded a motility rate significantly higher than that of the control, although at 7% *v*/*v*, it yielded the highest sperm motility (49.0 ± 6.5%; Figure 2a). With EG, the motility rates at 2% and 3% were significantly lower than that of the control (Figure 2b). Significantly lower motility was observed with all concentrations of PG as compared to the control, except with 7% *v*/*v*, where the difference was non-significant (Figure 2c). With Lac, no sperm motility was observed at any concentration; thus, the experiment was not repeated (Figure 2d). These results suggest that 7% *v*/*v* Glyc is the optimal low-Mw CPA.

### 3.3. Detailed Effect of CPLL

The highest motility rate after freeze–thawing was observed with 7% *v*/*v* Glyc (as the low-Mw CPA) and with 5% *w*/*v* CPLL (as the macro-Mw CPA). With CPLL, the lowest of the initially examined concentrations (i.e., 5–15% *w*/*v*) yielded the highest motility rate after freeze–thawing. Bovine studies have shown that CPLL can be effective at concentrations < 5% *w*/*v* [37]; therefore, CPLL concentrations from 0.05% to 5% *w*/*v* were evaluated. First, the same samples were subjected to concentrations ranging from 0.5% to 5% *w*/*v* CPLL, and no significant differences were observed between any concentration and the control (Figure 3a). Second, concentrations ranging from 0.05% to 0.5% *w*/*v* CPLL were investigated using the same samples. Here, a significant difference was observed between 0.3% *w*/*v* CPLL and the control, and the motility rate was 53 ± 8% (Figure 3b). These results indicate that 0.3% *w*/*v* CPLL may be the optimal macro-Mw CPA in motility. No saccharide was added to the cryopreservation reagents in all the examinations.

### 3.4. Effect of Saccharides

Saccharides can increase the osmotic pressure and viscosity of a carrier solution and promote vitrification of the external solution. Therefore, the saccharides’ ability to further improve the performance of the cryopreservation reagent was investigated. No significant differences were observed between cryopreservation reagents containing 0.05–0.5 M saccharides and the control (Figure 4a–c). Moreover, no significant differences were observed between cryopreservation reagents containing 0.05–0.3 M Raf and the control (Figure 4d). Raf did not dissolve at concentrations >0.4 M. The average sperm motility after freeze–thawing tended to be the highest at 0.3 M Fru, 0.3 M Suc, 0.3 M Tre, and 0.1 M Raf.

The differences in sperm motility between the saccharides under the aforementioned optimal conditions were also examined (*n* = 8). All the groups, including the control, were compared in the study. This analysis was performed after 3–4 weeks of cryopreservation. In the absence of an added saccharide, sperm motility was not observed after thawing. Moreover, although a significant difference was observed between Tre (17 ± 12%) and Raf (36 ± 9%), no significant differences were noted between Fru (25 ± 14%), Suc (25 ± 13%), and the control (23 ± 7%) (Figure 5a). Specifically, there was no significant difference between the control and each saccharide. These results suggest that the addition of a saccharide to the cryopreservation reagent is essential, and that including Tre may not be ideal for sperm motility after freeze–thawing.

### 3.5. Analysis of the Sperm Viability Test (SVT) and DNA Fragmentation

The remainder of the samples (*n* = 8) in Figure 5a was used to analyze viability, SVT and SDF. A significant difference in viability was observed between the free (i.e., ‘without saccharides added;’ 1 ± 1%) and non-free (i.e., ‘with saccharides added’) groups, and between Tre (40 ± 16%) and Raf (60 ±9%); however, no other significant differences were observed between the groups (Fru: 45 ± 11%, Suc: 49 ± 12%, control: 47 ± 15%; Figure 5b). Without saccharides, most sperms lacked both viability and motility. However, no significant differences in SDF were observed between the groups (Free: 32.7 ± 30.1%, Fru: 25.3 ± 28.5%, Suc: 12.7 ± 6.9%, Tre: 17.3 ± 7.4%, Raf: 8.2 ± 6.1%, control: 14.6 ± 7.8%; Figure 5c).

### 3.6. Quantitative Analysis of Sperm Motility with a Longevity Curve, Capacitation Status, and Acrosome Reaction

Sperm motility based on longevity, capacitation status, and acrosome reaction was compared between the developed cryopreservation reagent (containing 7% *v*/*v* Glyc, 0.3% *w*/*v* CPLL, and 0.1 M Raf; xeno) and the commercial reagent. Analysis of sperm motility with longevity by two-way repeated measures ANOVA showed no significant difference between the xeno and the control (Figure 6a). Moreover, no significant difference in the sperm capacitation status was observed between the xeno and the control (xeno, 26 ± 26%; control, 27 ± 23%; Figure 6b). Furthermore, the acrosome reaction was analyzed by flow cytometry using fluorescein isothiocyanate-conjugated peanut agglutinin (FITC-PNA), and its living/dead status was assessed using Ethidium Homodimer-1 (EthD-1). There was no significant difference in the acrosomal reaction between the xeno and the control (xeno, 73 ± 27%; control, 77 ± 21%; Figure 6c).

## 4. Discussion

We developed a new xeno-free and defined cryopreservation reagent for human sperms that lacked components derived from animals and HSA. Compared with commercial reagents, the new cryopreservation reagent did not induce any significant differences in sperm motility and viability based on longevity, capacitation status, and acrosome reaction after freeze–thawing. Additionally, performance similar to that reported by Zavos et al. was observed with 7% *v*/*v* low-Mw CPA Glyc [43]. This newly developed cryopreservation reagent is expected to address various problems, such as differences in lot quality, supply instability, zoonoses, and allergic reactions [44].

SCPT requires two types of CPAs: low-Mw CPAs, which permeate cell membranes to prevent intracellular ice crystal formation, and macro-Mw CPAs, which cannot permeate cell membranes and prevent extracellular ice crystal formation. In this study, we focused on macro-Mw CPAs which contained components derived from heterologous or human sources. TYB [43,45], HSA [46], HPC [23], and CPLL [37] are macro-Mw CPAs that have been used in previous studies. Among these, HPC [23] and CPLL [37] were the focus of this study. In the concentrations of macro-Mw CPAs, HPC and CPLL were 5–10% *w*/*v*, while those of TYB and serum were 20% *v*/*v* [47] in the previous report. Therefore, CPLL (5–15% *w*/*v*) was examined in this study. Moreover, the initial evaluation revealed the usefulness of 5% *w*/*v* CPLL (Figure 1). Accordingly, this component was further examined in detail using concentrations ≤ 5% *w*/*v*. We observed the highest sperm motility after freeze–thawing at 0.3% *w*/*v* CPLL (Figure 3). Currently, in bovine samples, the effective macro-Mw CPA CPLL concentration is ≤ 5% *w*/*v* [37]. In this study, we demonstrated that macro-Mw CPAs at concentrations < 5% *w*/*v* are also useful in human samples. However, although it is unclear why the motility rate decreased when the CPLL concentration was ≥5% *w*/*v*, embryo studies have shown that the optimum concentration of CPLL differs depending on the species [36].

Glyc [43,48], EG [48], PG [49], DMSO [48,49], acetamide [50,51,52], Lac [50], and formamide [53] have been reported as low-Mw CPAs; we used Glyc, EG, PG, and Lac in this study due to their low cytotoxicity to sperms. For human spermatozoa, 7% *v*/*v* Glyc has been reported as the optimal low-Mw CPA [43]. It has also been reported that the use of CPLL in bovines can reduce the concentration of Glyc [37]. Therefore, we investigated whether the use of CPLL could reduce the concentration of Glyc in this study as well. We observed that the Glyc concentration could not be reduced for unknown reasons (Figure 2).

Furthermore, we examined saccharides, including Suc, Tre, Raf [54,55], and Fru [55,56], which regulate osmotic pressure, thereby protecting the cell membrane. Regarding saccharides, there was no significant difference between the different concentrations of the same saccharides (Figure 4); however, there was a significant difference between Tre and Raf when compared between sugars with optimized concentrations (Figure 5a). Additionally, we observed that saccharides should be added for long-term cryopreservation (Figure 5a). Suc, Tre, and Raf have been proven effective when used in combination with HSA [54]. In a murine model, Raf was shown to be effective not only in combination with low-Mw or macro-Mw CPAs, but also when used alone [55]. The exact reason for this is unknown as there are no reports suggesting that saccharides are important for long-term storage of human sperms; it might be related to the saccharides’ protection of the cell membrane. Therefore, it was suggested that Fru, Suc, and Raf as saccharides may be important for long-term cryopreservation of human sperms.

We considered that an evaluation of motility only after freeze–thawing with the developed cryopreservation reagents would be insufficient. In recent years, SDF has been suggested to affect the outcomes of IVF, and cryopreservation is known to dramatically affect sperm physiology, such as its viability, motility, and chromatin integrity [57,58,59,60,61]. Accordingly, we analyzed sperm viability and SDF using a BD cell viability kit and the TUNEL assay, respectively. We used positive and negative controls and assessed validity of the analysis protocol (data not shown). Tre was associated with a significantly lower viability rate than Raf in cryopreservation reagents containing 7% *v*/*v* Glyc and 0.3% *w*/*v* CPLL (Figure 5b). The TUNEL assay revealed no significant difference in SDF (Figure 5c), and the fragmentation rates were within the expected range because DGC was performed prior to freezing. Furthermore, the developed cryopreservation reagent had the capacitation status and acrosome reaction comparable to that of the commercial reagent (Figure 6a–c).

In this study, we successfully developed a novel cryopreservation reagent; nonetheless, this study has a few limitations that should be considered. First, we achieved this goal by focusing on CPLL; however, the effects of other macro-Mw CPAs other than HPC and CPLL remain unclear. Second, we planned to investigate fertility after freeze–thawing. A test using a hamster model was planned to confirm fertility; however, such tests are tightly regulated in Japan. Therefore, the capacitation status (CTC/H258 stain) and acrosome reaction (FITC-PNA/EthD-1) were assessed in vitro. Acrosome reactions tend to account for a higher proportion of capacitation in sperms after freezing and thawing, with similar results reported in horses, bovines, and stallions [61,62]. Thirdly, the control results were very different. This study also included samples stored in an incubator at 37 °C, 5% CO_2_, and 5% O_2_ for 4–5 h until clinical IVF was completed.

Our results indicated no difference between the developed cryopreservation reagent and the commercial cryopreservation reagent. However, the effects on fertilization and subsequent embryo development could not be explored; thus, further investigations are required. Additionally, it is quite difficult to predict fertilizing ability of the sperm without an actual test as there are many more functional aspects of fertilization than just capacitation and the acrosome reaction. Moreover, the motility rates associated with freezing reagents containing TYB are widely known to be as high as approximately 70%. In clinical IVF, however, the risk of infection is prioritized, and cryopreservation reagents containing HSA, which show a motility rate of about 40%, are used. Although the new cryopreservation reagents we developed are clinically useful, there is room for improving the motility rate.

## 5. Conclusions

We developed a novel xeno-free and defined human sperm cryopreservation reagent that yielded motility, viability, and SDF similar to or better than those associated with commercial reagents containing HSA. Particularly, we demonstrated that the addition of macro-Mw CPAs at a low concentration is effective. This novel cryopreservation reagent may overcome the problems associated with TYB (i.e., zoonoses and allergic reactions) and HSA (i.e., allogeneic infections and unstable supplies due to variability between lots). The potential application of this newly developed cryopreservation reagent on sperms of other animal species warrants further investigation.

## Figures and Tables

**Table 1:** Concentrations used in each experiment, number of samples, and analysis method.

	Reageants		Concentrations	Units	*n*	Evaluation Item—Statistical Analysis
Figure 1	low-Mw CPAs	Glyc	4	% *v*/*v*	6	Motility—Dunnett’s test
	macro-Mw CPAs	HPC	5, 10, 15, 20	% *w*/*v*		
		CPLL	5, 7.5, 10, 12.5, 15			
	saccharides	—	—	—		
Figure 2	low-Mw CPAs	Glyc	2, 3, 4, 5, 6, 7, 8	% *v*/*v*	9	Motility—Dunnett’s test
		EG	2, 3, 4, 5, 6, 7, 8		9	
		PG	4, 5, 6, 7, 8, 9, 10		9	
		Lac	2, 3, 4, 5, 6, 7, 8,	% *w*/*v*	1	
	macro-Mw CPAs	CPLL	5	% *w*/*v*	/	
	saccharides	—	—	—	/	
Figure 3	low-Mw CPAs	Glyc	7	% *v*/*v*	/	Motility—Dunnett’s test
	macro-Mw CPAs	CPLL	0.05, 0.1, 0.2, 0.3, 0.4,0.5, 1, 2, 3, 4, 5	% *w*/*v*	18	
	saccharides	—	—	—	/	
Figure 4	low-Mw CPAs	Glyc	7	% *v*/*v*	/	Motility—Dunnett’s test
	macro-Mw CPAs	CPLL	5	% *w*/*v*	/	
	saccharides	Fru	0.05, 0.1, 0.2, 0.3, 0.4, 0.5	M	9	
		Suc	0.05, 0.1, 0.2, 0.3, 0.4, 0.5		9	
		Tre	0.05, 0.1, 0.2, 0.3, 0.4, 0.5		9	
		Raf	0.05, 0.1, 0.2, 0.3		9	
Figure 5	low-Mw CPAs	Glyc	7	% *v*/*v*	8	Motility—Tukey’s multiple comparison test Viability—Tukey’s multiple comparison test SDF—Tukey’s multiple comparison test
	macro-Mw CPAs	CPLL	5	% *w*/*v*		
	saccharides	Fru	0,3	M		
		Suc	0,3			
		Tre	0,3			
		Raf	0,1			
Figure 6	low-Mw CPAs	Glyc	7	% *v*/*v*	9	Quantitative analysis of sperm motility with a longevity curve—Two way–repeated measures analysis Capacitation status—Mann–Whitney U test Acrosomal reaction—Mann–Whitney U test
	macro-Mw CPAs	CPLL	5	% *w*/*v*		
	saccharides	Raf	0,1	M		
					105	

Glyc, glycerol; EG, ethylene glycol; PG, propylene glycol; LA, lactamide; HPC, hydroxypropyl cellulose; CPLL, carboxylated poly-l-lysine; Fru, fructose; Suc, sucrose; Tre, trehalose; Raf, raffinose; low-Mw CPAs, low-molecular-weight cryoprotective agents; macro-Mw CPAs, macromolecular-weight cryoprotective agents.

## Data Availability

The date supporting the findings of this study are available from the corresponding author upon request.

## Supplementary Materials

The following are available online at https://www.mdpi.com/article/10.3390/cells10061435/s1, Table S1: Parameters of raw semen and sperms after density gradient centrifugation (DGC) treatment, Table S2: Detailed parameters of raw semen and sperms after density gradient centrifugation (DGC) treatment.
